# Impact of Semaglutide as Weight Management Medication on Clinical Parameters and Health-Related Quality of Life: A Single-Center Study from Saudi Arabia

**DOI:** 10.3390/healthcare14070845

**Published:** 2026-03-26

**Authors:** Faten F. Bin Dayel, Rakan J. Alanazi, Miteb A. Alenazi, Sahar Alkhalifah, Dalal F. Bin Dayel, Wedad Mawkili, Abdulrahman Alwhaibi

**Affiliations:** 1 Department of Pharmacology and Toxicology, College of Pharmacy, Prince Sattam bin Abdulaziz University, Al-Kharj 11942, Saudi Arabia; f.bindayel@psau.edu.sa; 2 Pharmacy Practice Department, College of Pharmacy, Alfaisal University, Riyadh 11533, Saudi Arabia; rjalanazi@alfaisal.edu; 3 Department of Pediatrics, College of Medicine, King Saud University, Riyadh 11451, Saudi Arabia; mitalanazi@ksu.edu.sa; 4 Pharmacy Department, Imam Muhammad Ibn Saud Islamic University, Riyadh 13317, Saudi Arabia; saalkhalifa@imamu.edu.sa; 5 Department of Pharmaceutical Care, King Fahad Medical City, Riyadh 11316, Saudi Arabia; dbindayel@kfmc.med.sa; 6 Department of Pharmacology and Toxicology, College of Pharmacy, Jazan University, Jazan 45142, Saudi Arabia; wmawkili@jazanu.edu.sa; 7 Department of Clinical Pharmacy, King Saud University, Riyadh 11451, Saudi Arabia

**Keywords:** semaglutide, clinical parameters, weight, body mass index, quality of life

## Abstract

**Background:** Despite the cardiometabolic benefit of semaglutide, its impact on quality of life and whether patients’ characteristics influence clinical outcomes and health-related quality of life (HRQoL) remain ambiguous. **Method:** A retrospective review of patient charts was conducted after semaglutide initiation to assess the clinical impact of semaglutide, followed by a prospective analysis to evaluate HRQoL using the 36-Item Short Form Health Survey (SF-36). Descriptive and correlative analyses were conducted using SPSS software version 29 (IBM Corp., Armonk, NY, USA). **Results:** From a total of 715 patients, 255 (average age 59.1 years; 58.1% male participants) were subjected to clinical outcome analysis. The use of semaglutide was associated with significant reductions in HbA1c, total bilirubin, and TG and elevations in T4, TSH, Scr, and HDL. When each fifth value of each clinical parameter was compared with the baseline, gender revealed a significant impact, as females showed increased rates of elevated HDL (73.2% vs. 55.7%), reduced weight (69.8% vs. 55.7%), and reduced BMI (72.5% vs. 53.8%) compared to those in males. Despite the number of comorbidities significantly influencing BMI (*p* = 0.015), it had no impact on HbA1c post semaglutide use (*p* = 0.062). The same number of patients (n = 255), albeit having slightly different demographic and clinical characteristics, was included in the HRQoL analysis cohort. Females represented 54.5% of the cohort, and 71.0% were aged between 40 and <65 years. The average scores for all domains within the physical component summary (PCS) and mental component summary (MCS) were below 50, indicating a lack of perceived improvement in the overall quality of participants’ lives considering the pre-treatment period as the basis of comparison. In particular, younger age [OR 0.975, CI95% 0.953–0.998, *p* = 0.033] and being female [OR 0.273, CI95% 0.162–0.459, *p* < 0.001] led to reduced odds of scoring ≥ 50 in PCS, indicating a poor physical health state. On the contrary, older age [OR 1.036, CI95% 1.011–1.06, *p* = 0.004] increased the odds of scoring ≥ 50 in MCS, indicating a better mental health state in elderly vs. young semaglutide users. Although education level had significant influence on PCS, this did not extend to MCS. Upon investigating if type of change in a clinical parameter correlates with PCS and MCS scoring, only the decline in T4 reduced the odds of scoring MCS ≥ 50 [OR 0.5, CI95% 0.274–0.913, *p* = 0.024], while no significant influence was found either with other parameters or between clinical parameters and PCS. **Conclusions:** A lack of perceived improvement in HRQoL is noted with semaglutide use. Age, gender, and education play significant roles in HRQoL post semaglutide initiation. Overall, before prescribing semaglutide, patient counseling on its positive and negative effects is crucial to promote long-term adherence and optimize clinical outcomes.

## 1. Introduction

Semaglutide is a glucagon-like peptide 1 receptor (GLP-R1) agonist shown to improve glucose levels and reduce the risk of major adverse cardiovascular events [1,2]. It functions as an incretin mimetic by binding to the GLP-1 receptors in pancreatic β- and α-cells, the gastrointestinal tract, the central nervous system, the heart, and the kidneys [3,4]. This leads to enhanced glucose-dependent insulin secretion, reduced inappropriate glucagon release, delayed gastric emptying, and modulation in central pathways involved in appetite and satiety. Semaglutide was designed for once-weekly administration owing to its long-acting, fatty acid-conjugated structure that significantly prolonged its half-life [5,6]. Given these advantages, semaglutide was granted United States Food and Drug Administration (FDA) approval for use for type 2 diabetes mellitus (T2DM) treatment in December 2017.

Semaglutide’s clinical relevance has expanded since then, given its broader physiological impact, including modulation of hypothalamic pathways that regulate satiety, enhancement of insulin sensitivity in peripheral tissues, and reduction in inflammatory markers implicated in metabolic dysfunction [7,8,9]. An improvement in metabolic-associated fatty liver disease, a common comorbidity among individuals with T2DM and obesity, was evident in the ESSENCE trial without worsening fibrosis [10,11]. Beyond its role in blood glucose regulation and reductions in cardiovascular risk and metabolic dysfunction-associated steatohepatitis (MASH), semaglutide demonstrated a strong impact on obesity, supported by robust effects on appetite and satiety signaling [12,13]. Evidence from the STEP clinical trial program and multiple meta-analyses revealed that once-weekly semaglutide, especially at a dose of 2.4 mg, led to a substantial and sustained weight reduction in adults with and without T2DM [14]. Across randomized controlled trials and meta-analyses, semaglutide consistently achieved mean weight loss exceeding 10–15% of baseline body weight [14,15]. In addition to weight reduction, the use of semaglutide was significantly associated with an improvement in key clinical and cardiometabolic parameters including an increase in HDL cholesterol with a reduction in systolic and diastolic blood pressure, glycosylated hemoglobin A1C (HbA1C), fasting blood glucose, total cholesterol, LDL cholesterol, triglycerides, and C-reactive protein, as well as decreased BMI and waist circumference [14,16]. It is noteworthy that guidelines indicate that achieving 5–10% weight loss is clinically meaningful for improving metabolic function and reducing obesity-related complications. Additionally, a 5% reduction in body weight has been shown to significantly enhance multi-organ insulin sensitivity, while 5–10% weight loss has been associated with an approximately 0.6–1.0% decrease in HbA1C levels [17].

Although clinical indicators such as HbA1C, lipid profile, and BMI are essential for objectively evaluating the clinical effects of anti-diabetes and anti-obesity therapies, they do not fully reflect how these conditions and treatments affect patients’ daily lives. Obesity and T2DM influence physical functioning, mood, self-image, and social roles, all of which shape health-related quality of life (HRQoL). It has been shown that greater obesity severity and poor glycemic control were each independently linked to lower HRQoL, even after adjusting for comorbidities [18,19]. Relying only on clinical parameters therefore provides an incomplete picture of treatment benefit. Having said that, including patient-reported outcome measures (PROMs) would help in capturing these broader effects. For example, in the STEP 1–4 trials, semaglutide not only improved metabolic parameters and reduced body weight but also significantly enhanced physical functioning and overall HRQoL [20]. These findings highlight that pharmacologic interventions can improve both biological endpoints and patients’ perceived well-being and daily functioning.

While RCTs offer strong internal validity, their controlled settings and strict eligibility criteria often limit generalizability, particularly for patients with multiple comorbidities or from different cultural backgrounds [21]. In contrast, real-world data (RWD) studies further assess tolerability and quality-of-life outcomes in more diverse populations, offering a more holistic view of therapeutic impact [22]. Embedding HRQoL assessment within real-world research is therefore key for understanding the true, patient-centered benefits from treatments such as semaglutide.

To evaluate HRQoL in our cohort, we used the Short Form Health Survey questionnaire (SF-36), translated into an Arabic version and tailored to Saudi populations [23,24]. This version maintains the original eight domains of SP-36: physical functioning (PF), role limitations due to physical health (PR), bodily pain (BP), general health perceptions (GH), vitality [i.e., energy/fatigue] (VT), social functioning (SF), role limitations due to emotional problems (RE), and mental health (MH). Previous work has proven SF-36 to have good internal consistency and construct validity in patients with chronic diseases like diabetes [25]. In this study, SF-36 provides multi-dimensional insight into how patients perceive changes in both physical and mental health through semaglutide use.

Despite extensive semaglutide use in Saudi Arabia, there is a lack of research using culturally adapted tools to thoroughly assess both its clinical effects and how it impacts quality of life in real-world settings. Most regional studies have taken a one-dimensional approach focusing only on glycemic control and weight change, without considering patient functionality such as vitality, emotional role functioning, and social participation [26,27,28]. In addition, studies measuring HRQoL included an inadequate sample size or did not report the correlation of these outcomes with detailed clinical information [29,30]. By integrating clinical and quality-of-life measurements via SP-36, this study will achieve a more detailed and culturally appropriate representation of the benefits and limitations of semaglutide use in Saudi Arabia.

## 2. Methods

### 2.1. Study Design

This study was divided into two components. The first component was an observational retrospective review of patient charts to assess the clinical impact of semaglutide [quality of care analysis (QoC)]. The second component was a prospective evaluation of health-related quality of life (HRQoL) after semaglutide initiation. The pre-treatment period was used as the basis of comparison in both QoC and HRQoL. HRQoL was assessed through a structured telephone call followed by a text reminder and access to the 36-Item Short Form Health Survey (SF-36). 

### 2.2. Setting and Participants

**Figure 1:**
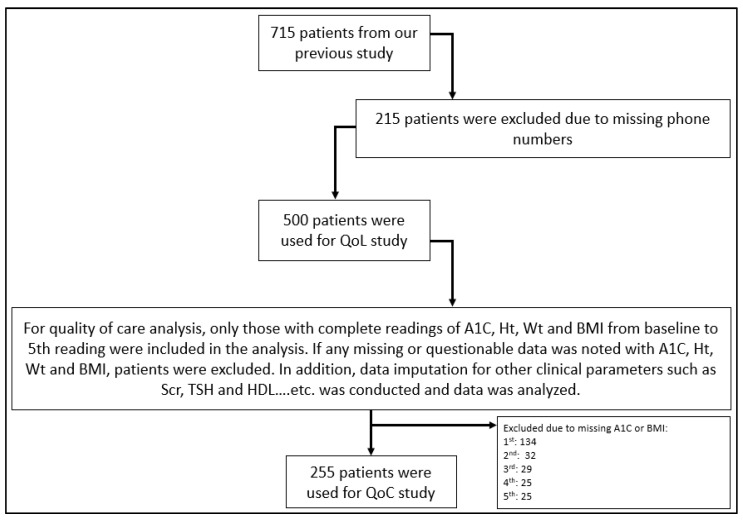
Demonstration of study design for assessing quality of care (n = 255).

**Figure 2:**
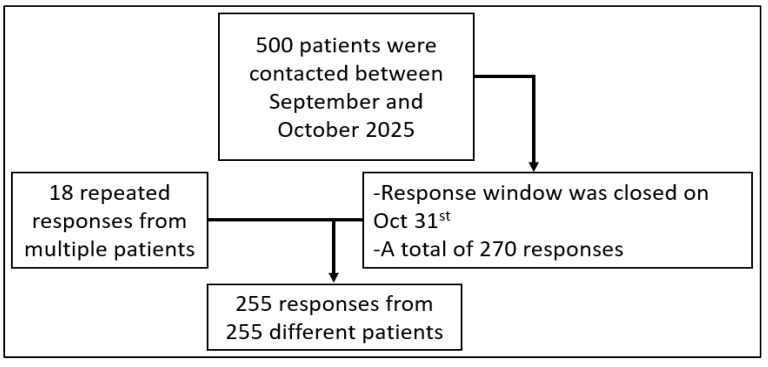
Demonstration of study design for quality of life (n = 255).

The study was conducted at King Saud University Medical City in Riyadh, Saudi Arabia, after receiving Research Project approval No. E-25-10081 from the institutional review board (IRB) of King Saud University Medical City on 1 September 2025. The initial study population was based on our previous study comprising 715 adults aged 18 years or older who were prescribed semaglutide between April 2022 and December 2024 [31]. For the retrospective component, we only included patients with complete documentation of HbA1c, height, weight, and body mass index (BMI) at baseline and all four follow-up visits, as well as a documented phone number. Four hundred and sixty were excluded, yielding a total of 255 participants to be included in the final QoC analysis (Figure 1). For the second prospective component, patients with a documented phone number that could be reached were eligible for inclusion. After removing duplicate responses, a total of 255 patients were included in the final HRQoL analysis (Figure 2).

### 2.3. Retrospective Data Collection

Demographic characteristics including age, gender, weight, BMI, and comorbidities were obtained from medical records. In addition, the following laboratory parameters were obtained: HbA1c, serum triglycerides (TG), high-density lipoprotein (HDL), low-density lipoprotein (LDL), total cholesterol, total bilirubin, thyroid-stimulating hormone (TSH), thyroxine (T4), and serum creatinine (Scr) at baseline and following four routine visits post semaglutide initiation. The change in each clinical parameter was evaluated in two ways: (a) longitudinally across all five documented readings, and (b) by comparing the baseline against the fifth reading. Additionally, for categorical analyses, changes in clinical parameters were defined as a decrease, no change, or increase according to the changes noted when the baseline was compared to the fifth reading.

### 2.4. Prospective Quality-of-Life Assessment

Quality of life was measured prospectively using a culturally adapted and validated Arabic version of SF-36 prepared for use in Saudi populations [32,33]. There are 8 domains evaluated in the SF-36 questionnaire including physical functioning (PF), role limitation because of physical health (RP), vitality (VT), mental health (MH), role limitation because of emotional problems (RE), bodily pain (BP), social functioning (SF) and general health (GH). The scores of items in each of the 8 domains range from 0 (the worst imaginable health state) to 100 (the best imaginable health state). However, the pre-coded responses of items in each domain are different. To make an accurate comparison between domains, the T-score transformation was performed for each domain score after adjustment by the population mean and standard deviation to produce norm-based scores with a common mean of 50 and standard deviation of 10. With that, the results of each domain can be meaningfully compared with others, and these scale scores can be directly interpreted in relation to the distribution of scores in the 2009 U.S. general population. The physical component summary (PCS) and mental component summary (MCS) were estimated based on all domains and adjusted by the population mean and standard deviation to produce norm-based scores with a common mean of 50 and standard deviation of 10. Another way to calculate PCS and MCS is to take the average of the 4 domains of PCS (PF-RP-BP-GH) and MCS (MH, EP, VT, SF). Overall, any PCS or MCS score below 50 represents a decrement from “normal” health and functioning, while scores ≥ 50 indicate improvement in health and functionality. How the T-score and PCS and MCS scores were calculated is explained in detail in the survey’s user manual [28].

Given the impact of semaglutide on the gastrointestinal system, GI symptoms were assessed using Yes/No questions related to the incidence of GERD, nausea/vomiting, constipation, diarrhea, flatulence, and dyspepsia after using semaglutide. Each GI item was scored as 100 if it was not experienced, and 0 if it was experienced. Since SF-36 did not include a domain related to GI, it was not possible to calculate the T-score, and simple scoring (out of 100) was used for analysis and comparison. Overall, the GI score ranges from 0 (the worst imaginable health state due to GI symptoms) to 100 (the best imaginable health state); thus, a higher average score indicates better health.

### 2.5. Statistical Analysis

Descriptive statistics were adopted for data analysis. Frequencies and percentages were used for categorical analyses, and a chi-squared test was used for associations between demographic data and changes in clinical parameters. Distribution normality of data was checked using the Shapiro–Wilk test and Kolmogorov–Smirnov tests, and group comparison was conducted accordingly via non-parametric statistics (Mann–Whitney U test, Kruskal–Wallis tests) or parametric statistics (independent Student’s *t*-test, one-way ANOVA) for continuous variables. Multivariate and univariate logistic regressions were used to examine predictors of questionnaire domain scores and demographic data, and the odds ratio (OR) with a 95% confidence interval (95% CI) was used to describe this association. All statistical analyses were performed using SPSS software version 29 (IBM Corp., Armonk, NY, USA), and *p* < 0.05 was taken to indicate statistical significance.

## 3. Results

Of the 715 patients that were included in our previous study [31], 215 were excluded due to missing contact numbers, leaving 500 patients (69.9%) that were contactable for quality-of-life assessment. From that 500, only 255 patients with almost complete documentation of HbA1C, TSH, T4, serum creatinine, HDL, TG, total cholesterol, and bilirubin at baseline and four follow-ups post semaglutide administration were subjected to analysis of clinical parameters related to treatment efficacy and safety. More details about the study sample are provided in Figure 1. The average age of patients was 59.1 years, with male gender representing 41.6%. Most patients were overweight and obese, at 24.7% and 71.8%, respectively. Of these patients, 70.7% had at least three comorbidities, with diabetes, dyslipidemia, and hypertension presenting in the majority, at 98.8%, 86.7%, and 72.5%, respectively. All clinical parameters at baseline were within the normal range except for HbA1C, which was 7.9%. Further details related to baseline characteristics are provided in Table 1.

**Figure 3:**
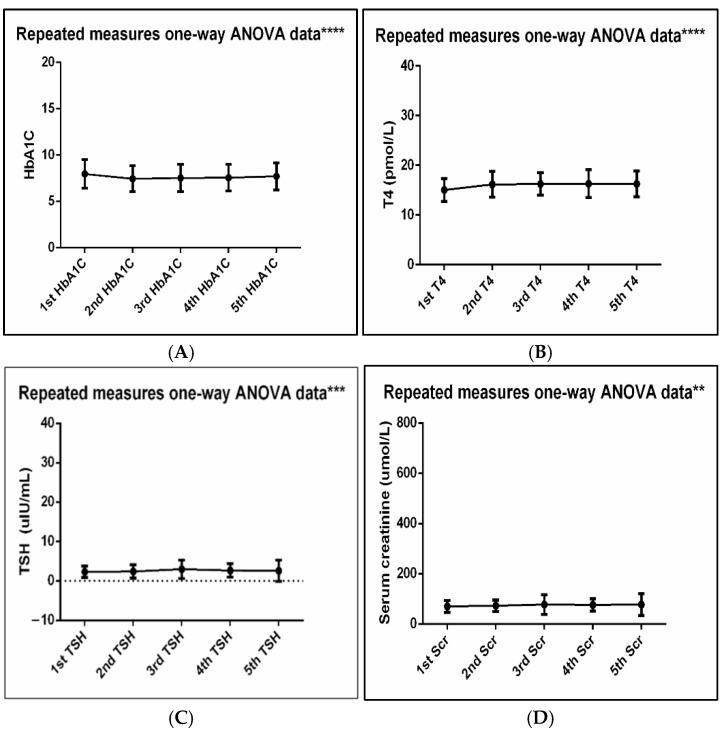

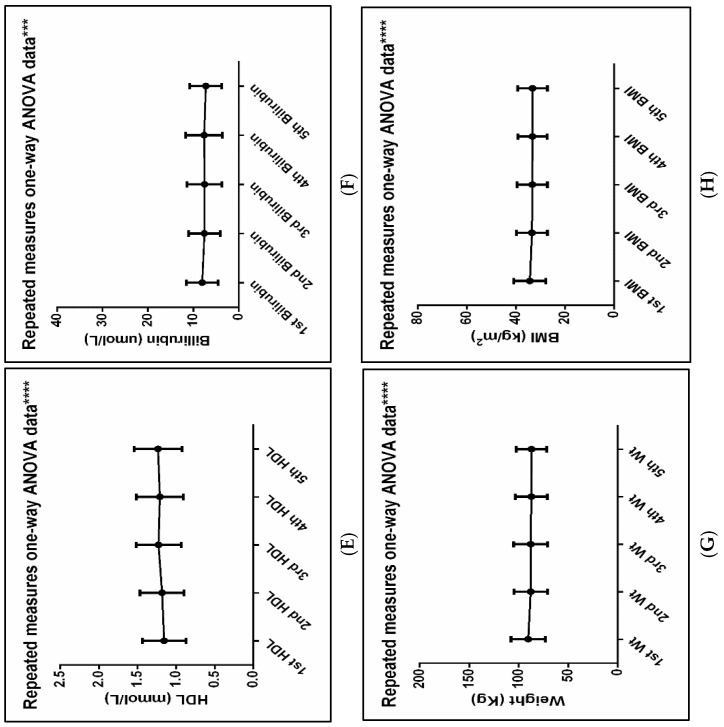
Comparison of clinical parameters that significantly changed at 5 points of care (n = 255). One-way ANOVA analysis of the baseline and all four follow-up visits readings of HbA1C (**A**), T4 (**B**), TSH (**C**), Scr (**D**), HDL (**E**), Bilirubin (**F**), weight (**G**) and BMI (**H**). ** *p* ≤ 0.01; *** *p* ≤ 0.001; **** *p* ≤ 0.0001.

Comparisons between clinical parameters at different monitoring timepoints are provided in Table 2 and Figure 3. Of these, HbA1C and bilirubin were significantly reduced, whereas T4, TSH, serum creatinine, and HDL were significantly elevated as patients continued treatment. Notably, weight and BMI also declined significantly as patients continued treatment. Further comparison of the baseline (first) and last (fifth) values revealed similar findings in addition to a reduction in triglycerides, as shown in Table 3. Further details about parameter findings at different timepoints are provided in Table 2 and Table 3.

To capture the association between demographic data (gender, age category, presence of comorbidities, and number of comorbidities) and the pattern of change in clinical parameters when the first and fifth checkup readings were compared, each fifth value of the clinical parameters was compared with the first value for every patient and it was determined whether it increased, decreased, or had not changed. On that basis, HDL, weight, and BMI appeared to be significantly affected by gender, at *p* = 0.012, *p* = 0.031, and *p* = 0.009, respectively, while HbA1C was not (*p* = 0.058), as shown in Table 4. In other words, female gender was significantly associated with an increased chance of elevated HDL (73.2% vs. 55.7%), reduced weight (69.8% vs. 55.7%), and lower BMI (72.5% vs. 53.8%) compared to male gender, while it tended to be associated with an increased chance of reducing A1C compared to male gender (60.4% vs. 45.3%). Notably, although having several comorbidities significantly influenced BMI (*p* = 0.015), their presence or absence tended to impact HbA1C (*p* = 0.062). When other clinical parameters were investigated for association with the same demographic variables, no significant association was observed. Further details are provided in Table 4. 

The SF-36 questionnaire was utilized with slight modification to assess the quality of life after initiation of semaglutide. Of the 500 individuals contacted, 255 responded, representing 51.0% of the contacted participants (Figure 2). Notably, this cohort differed slightly from the one used in the study’s first component in that only one hundred and twenty-three participants from the first component were included in the HRQoL evaluation. Age and the presence and number of comorbidities were significantly different between the two components of the study, while other baseline characteristics were very similar (Supplementary Table S1). Of those included in the HRQoL analysis, 54.5% were females, and most (71.0%) were aged between 40 and <65 years (Table 5). The average T-score of the questionnaire domains is 46.9, ranging between 43.9 and 49.1. PCS and MCS scores based on all eight domains were 47.3 and 46.8, respectively, demonstrating lack of perceived improvement among semaglutide-treated patients relative to normative values (Table 6). Overall GI scoring was calculated as 64.6 (Table 7). Patients were stratified based on age and gender, and all T-scores of domains as well as PCS and MCS were compared, as shown in Table 8 and Table 9. Based on age stratification, scores for emotional well-being and MCS based on all eight domains in the group aged ≥65 years were significantly higher than 50, indicating a better mental health state compared to young groups. Although role limitations due to emotional problems were significantly higher in the group aged ≥65 compared to that aged <40 years, the value was less than 50. The GI score was only significantly higher in the group aged ≥65 only vs. others (74.0 vs. 62.9 vs. 47.8 in ages ≥65, 40 to <65, and <40, respectively). According to gender stratification, all domains, with the exception of MCS, were significantly higher in males than females. Yet, only the BP and GH domains and PCS reflected better health compared to others, at 52.2, 50.3, and 50.4, respectively. Interestingly, unlike the age classification, there was no difference in GI symptom score between males and females.

Univariate logistic regression for PCS reveals that older age reduced the odds of scoring ≥ 50 in PCS [OR 0.975, CI95% 0.953–0.998, *p* = 0.033] and being female reduced the odds of scoring ≥ 50 in PCS compared to males [OR 0.273, CI95% 0.162–0.459, *p* < 0.001]. In other words, older age and being female were associated with a poor physical health state among semaglutide users (Table 10a). On the other hand, the odds of scoring ≥ 50 in PCS and a better physical health state are higher among bachelor-educated [OR 3.036, CI95% 1.388–6.643, *p* = 0.005] and doctorate-educated participants [OR 3.650, CI95% 1.349–9.876, *p* = 0.011] compared to elementary-educated participants. For MCS, older age [OR 1.036, CI95% 1.011–1.06, *p* = 0.004] increased the odds of scoring ≥ 50; semaglutide use was thus associated with a better mental health state in elderly compared to young users [OR 6.261, CI95% 1.592–24.616, *p* = 0.009]. However, unlike PCS, gender and education level had no impact on MCS (Table 10b). Regarding GI symptoms, older age increased the odds of scoring ≥ 50 in GI symptoms [OR 1.041, CI95% 1.014–1.069, *p* = 0.003], indicating that the elderly could tolerate GI symptoms and hence had a better health state (Table 11). Despite that, further investigation revealed that developing GI symptoms in general after semaglutide initiation reduced the odds of scoring ≥ 50 in both PCS and MCS, at [OR 0.431, CI95% 0.250–0.744, *p* = 0.002] and [OR 0.294, CI95% 0.167–0.517, *p* < 0.001], respectively, thereby reducing the perceived improvement in both physical and mental health.

Binary logistic regression analysis was conducted to determine whether net changes in lab parameters between the fifth and first readings (no change, increase, or decrease) had any impact on the odds for PCS or MCS scores (eight factors). Patients were stratified according to PCS and MCS to either < 50 or ≥50. Overall, the impact of parameter changes on PCS is negligible. Regarding MCS, however, a reduction in T4 was associated with reduction in the odds of scoring MCS ≥ 50 [OR 0.5, CI95% 0.274–0.913, *p* = 0.024]. In other words, the use of semaglutide was associated with a reduction in T4 that potentially predisposed semaglutide users to lower mental health. 

## 4. Discussion

In this research, the use of semaglutide was associated with both clinical and metabolic benefits, as shown in the laboratory and anthropometric parameters. Despite these benefits, patients showed a lack of perceived improvement in quality of life relative to normative values as reflected by all areas of the SF-36 quality-of-life survey. Notably, unlike the results reported in the SUSTAIN-6 trial [34], we found that the positive impact on laboratory parameters did not translate into improvement in the overall HRQoL. Cumulatively, focusing on the clinical outcomes only to evaluate efficacy of semaglutide is insufficient to capture the overall health benefit, and the inclusion of quality-of-life measures in conjunction with clinical outcomes provides better understanding of the overall impact of treatment on patients [35,36].

Our patients achieved notable improvements in several clinical metabolic parameters following semaglutide therapy. The levels of HbA1c (7.96% to 7.71%; *p* = 0.014), triglycerides (1.78 mmol/L to 1.63 mmol/L; *p* = 0.028), and bilirubin (8.04 µmol/L to 7.26 µmol/L; *p* < 0.001) were reduced, whereas HDL increased (1.15 mmol/L to 1.23 mmol/L; *p* < 0.001). Furthermore, reductions in weight (90.16 kg to 87.16 kg; *p* < 0.001) and BMI (34.41 kg/m^2^ to 33.21 kg/m^2^; *p* < 0.001) were detected. Our findings reiterate the benefit of using semaglutide in obese patients and those with metabolic disorders such as hypercholesterolemia and diabetes. This goes hand in hand with the results reported in the STEP 2 trial, as semaglutide 2.4 mg once weekly produced a superior, clinically meaningful reduction in body weight and improved glycemic control in adults with overweight and type 2 diabetes [37]. Similar findings on lipid profile were reported elsewhere, in which HDL increased and triglycerides decreased from baseline (adjusted estimated treatment differences at 12 months: +1.2 mg/dL HDL and −34.3 mg/dL triglycerides) [16]. Pooled randomized data also indicated that semaglutide improved lipid parameters in overweight and obese non-diabetic adults including reductions in total cholesterol, LDL, and triglycerides, with a modest increase in HDL [38].

It is noteworthy that not all participants responded similarly to treatment. Grouping patients based on their demographic data revealed a significant association of gender with HDL (*p* = 0.012), weight (*p* = 0.031), and BMI (*p* = 0.009). Females were more likely to have increased HDL levels and decreased weight and BMI compared to males. Age had a borderline effect on BMI changes (*p* = 0.078). Such results were reported in the SELECT trial, where women had a larger weight-loss difference in semaglutide vs. placebo treatment than men, and age-related differences were also observed [39]. We also found that subjects with more comorbidities have different BMI responses (*p* = 0.015), suggesting that BMI reduction varies with greater complexity of patient conditions. This is in line with evidence showing that initial patient characteristics affect the magnitude of weight response such that in overweight/obese adults without T2D, semaglutide 2.4 mg once weekly produced clinically meaningful weight-loss differences and improved glycemic control compared with those with T2D [40].

Intriguingly, a significant increase in thyroid-stimulating hormone (2.27 to 2.72, *p* = 0.019) and serum creatinine (70.54 to 77.79, *p* = 0.002) was associated with semaglutide use. Current data on the effects of GLP-1 agonists on thyroid function is not conclusive; the observed increase in TSH should therefore be considered hypothesis-generating and the findings must be interpreted in the context of pre-existing thyroid disease and levothyroxine use [41], concomitant medications and other co-existing illness. Based on kidney function, the available trials indicate that use of semaglutide is a kidney-protecting strategy in at-risk patients [42]. In the FLOW study, semaglutide significantly reduced major kidney events and cardiovascular death vs. a placebo in patients with type 2 diabetes mellitus and established chronic kidney disease [43]. In contrast, there have been rare case reports describing rapid worsening of renal function and acute kidney injury in patients with pre-existing CKD who experience adverse gastrointestinal symptoms after semaglutide use [44]. A slight increase in creatinine levels, which was observed in our participants, must therefore be interpreted in the context of baseline renal function, reversible causes including medications being used, and other patient comorbidities. This reflects the need for individualized renal follow-up for patients after semaglutide initiation or escalation of doses, especially for patients at higher risk such as CKD and diabetic patients.

Despite improvements in clinical parameters, a lack of improvement in several domains of quality of life, both physically and mentally, has been observed, which contradicts the results reported in the SUSTAIN 6 trial [34]. Such inconsistencies were reported in diabetes and obesity studies, where weight loss and improved glycemic control were not correlated with improvement in the total and psychological scores included in quality-of-life evaluation, despite the improvements in physical aspects, particularly in patients with other comorbidities [45,46,47]. Intriguingly, although the post hoc results of the STEP and SUSTAIN 6 clinical trials showed mild improvement in quality of life of semaglutide-treated patients, particularly in measured physical function compared to a placebo in the STEP trials, these benefits were not uniformly above population norms for physical and vitality domains, especially in older or multi-comorbid populations in SUSTAIN 6, where the observed effect sizes were limited and did not translate into clinical benefit across all SF-36 domains [1,20,48]. Our results align with these findings, where semaglutide administration was not associated with an optimal enhancement in quality of life.

Age has been identified as an outcome modifier regarding quality of life in our study. Although advanced age showed no consistent association with scores of the physical component summary, elderly (aged 65 years or older) patients reported higher mental health scores compared to younger patients. These results are not conflicting; rather, they illustrate the longstanding theories related to aging, as older people tend to experience gradual physical impairments due to the effects of sarcopenia, osteoarthritis, and cardiometabolic diseases, which may reduce their physical function despite improvements in their clinical parameters [49,50,51]. On the other hand, older people’s mental health may be sustained or even improved through emotional regulation, redefinition of health expectations, and calmer reactions to psychological distress, i.e., psychological adaptation. This has been shown in previous studies where older people’s perceived mental health status was equivalent or even superior to that of young people with a similar level of disease, a concept referred to as the ‘paradox of aging’ [52,53]. In addition, sociocultural habits specific to the regional area might contribute to psychological adaptation among the elderly despite their poor physical functioning. In other words, extended family structures and strong intergenerational relationships in Saudi Arabia are common, with older adults often remaining integrated within family households and community networks. Such social cohesiveness could potentially provide emotional stability and psychological resilience even in the presence of physical health challenges. Although social support variables were not directly assessed in this study, such interaction warrants further investigation in the future.

Another factor influencing our results is gender. According to gender stratification, all domains, with the exception of MCS, were significantly higher in males compared to females. Although positive effects on health-related quality of life, especially in physical function, were shown in the STEP and SUSTAIN 6 trials [20,34,48], the SF-36 questionnaire results were not reported with gender-stratified analyses. Additionally, besides women scoring lower values for most of the SF-36 domains in our study, they had lower odds of scoring PCS ≥ 50 compared to men, although they had greater changes in body weight and BMI, which is aligned with a recently published meta-analysis [54]. This could be explained through women experiencing a heavier symptom burden, increased pain sensitivity, and a greater sense of psychological distress than their male peers for a given level of disease severity. Currently, there is a lack of research on sex-specific patient outcomes regarding semaglutide treatment. However, the effects of gender [55], caregiver roles, and societal pressures could possibly influence the magnitude of symptoms experienced regardless of metabolic change [56]. This potentially explains why all domains of the SF-36 questionnaire were significantly higher (indicating a better health state) in men compared to women.

Besides age and gender, a significant correlation was found between thyroxine levels and changes in mental health. The role of thyroid hormones in regulating mood, processing information, and feeling emotionally well is well-established [57,58]. Even subtle variation in these hormones has been associated with depressive symptoms, fatigue, and reduced quality-of-life parameters [59]. Although semaglutide does not specifically target the hypothalamic–pituitary–thyroid axis, an indirect effect can occur through the weight-reducing properties of GLP-1 receptor agonists. However, in contrast to the reduced thyroid functions reported in observational studies [41], both T4 and TSH levels were elevated in our patients, implying an indirect endocrinopathic response rather than a class effect. Some observational studies have shown modest changes in thyroid hormone parameters associated with semaglutide treatment that were mainly attributed to the weight-reducing properties of semaglutide [60]. Overall, reduced mental well-being (reduction in the odds of scoring MCS ≥ 50) is linked to lower T4 levels in our population, which is in line with previous neuroendocrine relationships between thyroid hormone levels and neuropsychological function [61]. These findings, however, suggest the need for further investigation into the underlying mechanisms and do not establish causality.

Although the T-score was not calculated for gastrointestinal symptoms, the GI symptoms were perceived as less severe among elderly compared to younger people. While GI adverse effects are well documented and affect a substantial proportion of semaglutide users according to post-marketing surveillance and the FDA Adverse Event Reporting System [62], age-specific analyses of symptom perception and HRQoL impact remain limited in randomized trials. This could be interpreted as age-related changes in visceral sensitivity, gastric accommodation, and pain thresholds; whereas elderly patients may adapt to the symptoms and alter their expectations regarding discomfort, the younger generation may experience GI symptoms as being more disruptive to their daily functioning [63]. These findings suggest the necessity for GI symptom-related counseling to be age-tailored when semaglutide is indicated.

Several limitations exist in our study and should be considered prior to applying our findings to clinical practice. First, the single-centered and observational nature of the study limits the ability to generalize our findings, establish causative association between patient characteristics, and analyze the impact of treatment on clinical parameters and quality of life. Second, selecting participants with complete lab findings at four points of care after semaglutide initiation and a contact number led to an inclusion rate of 35.7% (255 of 715) for clinical parameter analysis. Third, only half of the candidates available for quality-of-life assessment completed the SF-36 questionnaire (255 of 500). Notably, this quality-of-life cohort (n = 255) is different from the quality-of-care cohort (n = 255) in the sense that it is significantly younger and has less participants with comorbidities (Supplementary Table S1). Consequently, this could predispose to selection bias and have influence on the scope of variables and interpretation of findings. Fourth, our interpretation of clinical parameters and quality of life lacks pharmacological validity since neither semaglutide dose nor treatment duration was collected for our participants. In addition, duration between follow-up visits was not documented, which again limits the generalizability of the results. However, as this study is the first to be conducted on our population, it should serve as the starting point for future longitudinal research to enrich the literature with more valid and reliable results. Despite these limitations, this study reflects real-world data collected at multiple timepoints (five measurements in total for each patient), which allowed us to evaluate the effects of intervention on these quality-of-life measures over time. Additionally, the use of an Arabic-language, easy-to-complete, validated version of the SF-36 quality-of-life survey yielded trustworthy findings that could be used for comparative purposes in future studies conducted on our population.

## 5. Conclusions

Despite the advantages of utilizing semaglutide for HbA1C, weight, BMI, and HDL, more attention should be paid to bilirubin, T4, TSH, and serum creatinine. Age, gender, and education level of patients played significant roles in quality of life after semaglutide initiation. Although reducing weight is one of the major goals of using semaglutide, the overall lack of improvement in quality of life should be considered prior to initiation in patients qualified for semaglutide treatment. Patient counseling regarding the positive and negative effects of semaglutide use is crucial prior to prescribing the medication to promote long-term adherence and optimize clinical outcomes.

## Figures and Tables

**Table 1:** Baseline characteristics for participants in clinical parameter assessment (n = 255).

Demographic Variable	
Age (years, mean years ± SD)	59.1 ± 9.7
BMI (kg/m^2^, mean years ± SD)	34.4 ± 6.6
Gender, n (%)	
Male	106 (41.6)
Female	149 (58.4)
BMI, n (%)	
Healthy weight	9 (3.5)
Overweight	63 (24.7)
Obesity	
Type I	76 (29.8)
Type II	65 (25.5)
Type III	42 (16.5)
Participant with comorbidities, n (%)	
Yes	252 (98.8)
No	3 (1.2)
DM, n (%)	252 (98.8)
DLD, n (%)	221 (86.7)
HTN, n (%)	185 (72.5)
Stroke, n (%)	5 (2.0)
CHF, n (%)	7 (2.7)
IHD, n (%)	40 (15.7)
A-fib, n (%)	5 (2.0)
MI, n (%)	2 (0.8)
CKD, n (%)	12 (4.7)
Number of comorbidities, n (%)	
0	2 (0.8)
1	12 (4.7)
2	61 (23.9)
3	133 (52.2)
4	40 (15.7)
5	6 (2.4)
6	1 (0.4)
HbA1C (%, mean years ± SD)	7.9 ± 1.5
T4 (pmol/L, mean years ± SD)	14.9 ± 2.6
TSH (uIU/mL, mean years ± SD)	2.4 ± 1.7
Serum creatinine (mcmol/L, mean years ± SD)	70.4 ± 24.2
HDL (mmol/L, mean years ± SD)	1.2 ± 0.3
LDL (mmol/L, mean years ± SD)	2.3 ± 0.9
TG (mmol/L, mean years ± SD)	1.8 ± 1.3
TC (mmol/L, mean years ± SD)	4.2 ± 1.0
Bilirubin (umol/L, mean years ± SD)	8.1 ± 3.6

**Table 2:** Comparison of initial clinical parameter readings vs. other follow-up readings (n = 255).

Parameter	Point of Care					*p*
	1st	2nd	3rd	4th	5th	
HbA1C *	7.96 ± 1.54	7.44 ± 1.41	7.53 ± 1.49	7.56 ± 1.42	7.71 ± 1.47	<0.001
T4 #	15.04 ± 2.38	16.10 ± 2.58	16.25 ± 2.36	16.40 ± 2.76	16.18 ± 2.53	<0.001
TSH #	2.27 ± 1.56	2.46 ± 1.87	2.96 ± 2.34	2.75 ± 1.83	2.72 ± 2.78	<0.001
Scr #	70.54 ± 24.21	72.79 ± 22.38	77.66 ± 39.77	75.90 ± 25.19	77.79 ± 43.40	<0.001
HDL #	1.15 ± 0.28	1.18 ± 0.28	1.23 ± 0.29	1.21 ± 0.30	1.23 ± 0.31	<0.001
LDL	2.28 ± 0.84	2.20 ± 0.83	2.27 ± 0.84	2.24 ± 1.05	2.19 ± 0.83	0.187
TG	1.78 ± 1.28	1.66 ± 0.82	1.69 ± 1.02	1.70 ± 1.43	1.63 ± 0.97	0.177
TC	4.24 ± 1.01	4.13 ± 0.97	4.24 ± 0.98	4.19 ± 1.18	4.17 ± 1.04	0.272
Bilirubin *	8.04 ± 3.44	7.55 ± 3.53	7.51 ± 3.85	7.63 ± 4.03	7.26 ± 3.52	<0.001
Wt *	90.16 ± 16.71	87.84 ± 17.16	87.91 ± 17.09	87.29 ± 16.07	87.16 ± 15.47	<0.001
BMI *	34.41 ± 6.55	33.61 ± 6.67	33.39 ± 25.0	33.22 ± 5.96	33.21 ± 6.03	<0.001

* Significant reduction in HbA1C (2nd, 3rd, and 4th vs. 1st only AND 5th vs. 2nd), bilirubin (2nd and 5th vs. 1st), weight (2nd, 3rd, 4th, and 5th vs. 1st), and BMI (2nd, 3rd, 4th, and 5th vs. 1st). # Significant elevation in T4 (2nd, 3rd, 4th, and 5th vs. 1st), TSH (3rd and 4th vs. 1st AND 3rd vs. 2nd), Scr (2nd, 3rd, 4th, and 5th vs. 1st AND 4th vs. 2nd), and HDL (3rd and 4th vs. 1st AND 2nd vs. 5th).

**Table 3:** Comparison of the 1st vs. 5th clinical parameters (n = 255).

Clinical Parameter (Mean ± SD)	1st Monitoring (n = 255)	5th Monitoring (n = 255)	*p* Value
HbA1C *	7.96 ± 1.54	7.71 ± 1.47	0.014
T4 #	15.04 ± 2.38	16.18 ± 2.53	<0.001
TSH #	2.27 ± 1.56	2.72 ± 2.78	0.019
Scr #	70.54 ± 24.21	77.79 ± 43.40	0.002
HDL #	1.15 ± 0.28	1.23 ± 0.31	<0.001
LDL	2.28 ± 0.84	2.19 ± 0.83	0.125
TG *	1.78 ± 1.28	1.63 ± 0.97	0.028
TC	4.24 ± 1.01	4.17 ± 1.04	0.379
Bilirubin *	8.04 ± 3.44	7.26 ± 3.52	<0.001
Wt *	90.16 ± 16.71	87.16 ± 15.47	<0.001
BMI *	34.41 ± 6.55	33.21 ± 6.03	<0.001

* Significant reduction in HbA1c, TG, bilirubin, Wt, and BMI. # Significant elevation in T4, TSH, Scr, HDL, and bilirubin; No significant change in LDL and TC.

**Table 4:** Association between clinical parameters that significantly changed or tended to change with gender, age category, presence of comorbidities, and total number of comorbidities (n = 255).

Clinical Parameters	Demographic Variable	No Change n (%)	Increased n (%)	Decreased n (%)	*p* Value
HbA1C	Gender				0.058
	Male	6 (5.7)	52 (49.1)	48 (45.3)	
	Female	6 (4.0)	53 (35.6)	90 (60.4)	
HDL	Gender				0.012
	Male	4 (3.8)	59 (55.7)	43 (40.6)	
	Female	2 (1.3)	109 (73.2)	38 (25.5)	
Weight	Gender				0.031
	Male	2 (1.9)	45 (42.5)	59 (55.7)	
	Female	5 (3.4)	40 (26.8)	104 (69.8)	
BMI	Gender				0.009
	Male	1 (0.9)	48 (45.3)	57 (53.8)	
	Female	1 (0.7)	40 (26.8)	108 (72.5)	
BMI	Age				0.078
	<40	0 (0.0)	1 (12.5)	7 (87.5)	
	40 to <65	0 (0.0)	67 (37.4)	112 (62.6)	
	≥65	2 (3.0)	20 (29.9)	45 (67.2)	
HbA1C	Comorbidities				0.062
	Yes	11 (4.4)	104 (41.3)	137 (54.4)	
	No	1 (33.3)	1 (33.3)	1 (33.3)	
BMI	Number of comorbidities				0.015
	0	0 (0.0)	1 (50.0)	1 (50.0)	
	1	0 (0.0)	4 (33.3)	8 (66.7)	
	2	0 (0.0)	20 (32.8)	41 (67.2)	
	3	0 (0.0)	46 (34.6)	87 (65.4)	
	4	1 (2.5)	14 (35.0)	25 (62.5)	
	5	1 (16.7)	2 (33.3)	3 (50.0)	
	6	0 (0.0)	1 (100.0)	0 (0.0)	

**Table 5:** Baseline characteristics of participants in QoL assessment (n = 255).

Demographic Variable	
Gender, n (%)	
Male	116 (45.5)
Female	139 (54.5)
Age (mean years ± SD) *	57.3 ± 11.1
Age classification, n (%)	
<40	15 (5.9)
40 to <65	181 (71.0)
≥65	59 (23.1)
Education, n (%)	
Elementary	42 (16.5)
Intermediate	19 7.5)
Secondary	59 (23.1)
Bachelor	85 (33.3)
Master	21 (8.2)
Doctorate	29 (11.4)

* Missing data.

**Table 6:** Score distributions of the SF-36 multi-item questionnaire and healthcare component summary [ranging from 0 (the worst imaginable health state) to 100 (the best imaginable health state)].

Description of the Multi-Item Domains	Number of Questions	T-Score (Mean ± SD)
Physical functioning (PF)	10	43.9 ± 13.1
Role limitations due to physical health (RP)	4	47.2 ± 11.6
Emotional well-being (MH)	5	45.6 ± 12.6
Role limitations due to emotional problems (RE)	3	47.1 ± 12.5
Energy/fatigue (VT)	4	47.2 ± 10.9
Social functioning (SF)	2	46.3 ± 11.4
Bodily pain (BP)	2	49.1 ± 11.1
General health (GH)	5	48.4 ± 9.7
Description of the Healthcare Component Summary	Number of Domains	T-Score (Mean ± SD)
Physical component summary (PCS)	8	47.3 ± 9.6
Mental component summary (MCS)	8	46.8 ± 12.2
Physical component summary (PCS)	4	47.2 ± 8.7
Mental component summary (MCS)	4	46.5 ± 9.9

**Table 7:** Occurrence and score of GI symptoms after semaglutide use.

Symptoms	Occurrence (n (%))	Score (Mean ± SD)
GERD	85 (33.3)	66.7 ± 47.2
Nausea and vomiting	100 (39.2)	60.8 ± 48.9
Constipation	113 (44.3)	55.7 ± 49.8
Diarrhea	66 (25.9)	74.1 ± 43.9
Flatulence or gas	94 (36.9)	63.1 ± 48.3
Dyspepsia	83 (32.5)	67.5 ± 46.9
Average of all symptoms, ranging from 0 (the worst imaginable health state) to 100 (the best imaginable health state)		64.6 ± 30.8

**Table 8:** Score distribution according to age and health domains.

Age	Less than 40 Years (n = 15)	40 to <65 Years (n = 181)	≥65 Years (n = 59)	*p* Value
Description of the Domains [T-Score (Mean ± SD)]				
Physical functioning (PF)	44.1 ± 18.0	44.7 ± 12.5	41.8 ± 13.5	0.350
Role limitations due to physical health (RP)	45.2 ± 13.4	47.4 ± 11.7	47.2 ± 11.0	0.776
Emotional well-being (MH) #	35.7 ± 13.5	44.9 ± 12.9	50.2 ± 9.4	<0.001
Role limitations due to emotional problems (RE) *	39.5 ± 15.6	47.1 ± 12.4	48.9 ± 11.2	0.032
Energy/fatigue (VT)	45.0 ± 12.8	46.5 ± 11.4	49.7 ± 8.8	0.112
Social functioning (SF)	41.3 ± 12.9	46.1 ± 11.4	48.3 ± 10.4	0.087
Bodily pain (BP)	52.7 ± 10.6	48.2 ± 11.4	50.8 ± 10.2	0.137
General health (GH)	50.7 ± 10.4	47.5 ± 9.9	50.4 ± 8.6	0.090
Physical component summary (PCS) 8 domains	51.9 ± 12.8	47.3 ± 9.0	46.1 ± 10.5	0.108
Mental component summary (MCS) 8 domains #	36.7 ± 14.3	46.1 ± 12.1	51.3 ± 9.9	<0.001
Physical component summary (PCS) 4 domains	48.2 ± 10.5	46.9 ± 8.7	47.5 ± 8.2	0.814
Mental component summary (MCS) 4 domains *	40.4 ± 11.2	46.2 ± 10.2	49.3 ± 7.8	0.005
**Description of the domain [score (mean ± SD)]**	**Less than 40 years** **(n = 15)**	**40 to <65 years** **(n = 181)**	**≥65 years** **(n = 59)**	***p*** **value**
GI symptoms ^	47.8 ± 35.6	62.9 ± 40.4	74.0 ± 28.2	0.005

# For each group vs. others; * for age group < 40 vs. age ≥ 65; ^ for age ≥ 65 only vs. others.

**Table 9:** Score distribution according to gender and health domains.

Gender	Male (n = 116)	Female (n = 139)	*p* Value
Description of the Domains [T-Score (Mean ± SD)]			
Physical functioning (PF)	47.9 ± 11.1	40.7 ± 13.8	<0.001
Role limitations due to physical health (RP)	49.6 ± 10.5	45.3 ± 12.2	0.003
Emotional well-being (MH)	47.8 ± 12.1	43.7 ± 12.7	0.009
Role limitations due to emotional problems (RE)	49.2 ± 11.3	45.4 ± 13.2	0.014
Energy/fatigue (VT)	49.1 ± 10.7	45.6 ± 11.0	0.010
Social functioning (SF)	48.3 ± 10.4	44.7 ± 11.9	0.011
Bodily pain (BP)	52.2 ± 9.5	46.5 ± 11.7	<0.001
General health (GH)	50.3 ± 8.7	46.7 ± 10.3	0.004
Physical component summary (PCS) 8 factors	50.4 ± 8.6	44.7 ± 9.8	<0.001
Mental component summary (MCS) 8 factors	48.2 ± 11.6	45.6 ± 12.4	0.083
Physical component summary (PCS) 4 factors	49.9 ± 7.7	44.8 ± 8.7	<0.001
Mental component summary (MCS) @ 4 factors	48.6 ± 9.4	44.8 ± 10.1	0.002
**Description of the Domains [Score (Mean ± SD)]**	**Male** **(n = 116)**	**Female** **(n = 139)**	***p*** **Value**
GI symptoms	67.5.8 ± 29.5	62.2 ± 31.7	0.171

**Table 10:** (a) Factors associated with PCS score (8 factors). (b) Factors associated with MCS score (8 factors).

**(a)**						
**Variable**		**Coef (B)**	**S.E.**	**Odds Ratio** **[Exp (B)]**	**95% CI** **Upper–Lower**	***p*** **Value**
Age *	Age in years	−0.025	0.012	0.975	0.953–0.998	0.033
Age group						0.304
	<40 yrs			1		-
	40 to <65	−0.527	0.548	0.590	0.202–1.727	0.336
	≥65	−0.853	0.591	0.426	0.134–1.356	0.149
Gender *	Male					<0.001
	Female	−1.299	0.265	0.273	0.162–0.459	
Education *						0.005
	Elementary			1		-
	Intermediate	−0.519	0.654	0.595	0.165–2.145	0.427
	Secondary	0.354	0.427	1.425	0.617–3.294	0.407
	Bachelor	1.111	0.399	3.036	1.388–6.643	0.005
	Master	0.707	0.550	2.028	0.690–5.958	0.198
	Doctorate	1.295	0.508	3.650	1.349–9.876	0.011
**(b)**						
**Variable**		**Coef (B)**	**S.E.**	**Odds Ratio** **[Exp (B)]**	**95% CI** **Upper–Lower**	***p*** **Value**
Age *	Age in years	0.035	0.012	1.036	1.011 −1.06	0.004
Age group						0.014
	<40 yrs			1		-
	40 to <65	1.198	0.663	3.313	0.904–12.139	0.071
	≥65	1.843	0.699	6.261	1.592–24.616	0.009
Gender *	Male					0.134
	Female	−0.379	0.253	0.685	0.417–1.124	
Education						0.722
	Elementary			1		-
	Intermediate	−0.634	0.576	0.530	0.174–1.612	0.263
	Secondary	−0.403	0.406	0.668	0.302–1.481	0.321
	Bachelor	−0.213	0.378	0.808	0.385–1.694	0.573
	Master	0.192	0.538	1.212	0.422–3.482	0.721
	Doctorate	−0.026	0.483	0.974	0.378–2.511	0.957

* Older age reduces the odds of scoring ≥ 50 in PCS and being female reduces the odds of scoring ≥ 50 in PCS compared to males; hence, older age and being female lead to a poor physical health state in semaglutide users. On the other hand, having bachelor- and doctorate-level education increases the odds of scoring ≥ 50 in PCS compared to elementary education, which leads to a better physical health state. * Older age increases the odds of scoring ≥ 50 in MCS, which leads to a better mental health state in elderly compared to young semaglutide users, while gender and education level have no such impact.

**Table 11:** Factors associated with GI scoring.

Variable		Coef (B)	S.E.	Odds Ratio [Exp (B)]	95% CI Upper–Lower	*p* Value
Age *	Age in years	0.040	0.014	1.041	1.014–1.069	0.003
Age group						0.083
	<40 yrs			1		-
	40 to <65	0.586	0.553	1.796	0.608–5.308	0.290
	≥65	1.309	0.639	3.704	1.508–12.970	0.041
Gender	Male					0.234
	Female	−0.350	0.294	0.704	0.396–1.254	

Patients were stratified according to the GI score to either <50 or ≥50 with prediction for score ≥ 50. Reference groups for age, age group, and gender. * Older age increases the odds of an average score ≥ 50 in GI symptoms, indicating a better health state.

## Data Availability

The original contributions presented in this study are included in the article. Further inquiries can be directed to the corresponding author.

## Supplementary Materials

The following supporting information can be downloaded at https://www.mdpi.com/article/10.3390/healthcare14070845/s1, Table S1. Comparison of demographic and baseline characteristics between the groups enrolled in the retrospective (QoC) and prospective cohort (HRQoL).
