# Supplementary material for: Impact of Semaglutide as Weight Management Medication on Clinical Parameters and Health-Related Quality of Life: A Single-Center Study from Saudi Arabia
Source: Healthcare (Basel). 2026 Mar 26;14(7):845. doi: 10.3390/healthcare14070845 (PMC13073150; doi:10.3390/healthcare14070845)
Supplement: Supplementary file 1 [file healthcare-14-00845-s001.zip › healthcare-4150396-supplementary.pdf]

Article

Supplementary Table S1. Comparison of demographic and baseline characteristics between the groups enrolled in the retrospective (QoC) and prospective cohort (HRQoL).

**Demographic variable**	**QoL (n=255)**	**QoC (n=255)**	**P value**
Age (years, mean years ± SD)	57.3 ± 11.1	59.1 ± 9.7	0.047
BMI (kg/m2, mean years ± SD)	34.8 ± 6.9	34.4 ± 6.6	0.510
Gender, n (%)			0.422
Male	116 (45.5)	106 (41.6)	
Female	139 (54.5)	149 (58.4)	
BMI, n (%)			0.913
Healthy weight	7 (3.0)	9 (3.5)	
Overweight	50 (21.7)	63 (24.7)	
Obesity			
Type I	72 (31.3)	76 (29.8)	
Type II	58 (25.2)	65 (25.5)	
Type III	43 (18.7)	42 (16.5)	
Participant with comorbidities, n (%)			< 0.001
Yes	213 (92.6)	252 (98.8)	
No	17 (7.4)	3 (1.2)	
DM, n (%)	213 (92.6)	252 (98.8)	< 0.001
DLD, n (%)	181 (78.7)	221 (86.7)	0.022
HTN, n (%)	153 (66.5)	185 (72.5)	0.166
Stroke, n (%)	5 (2.2)	5 (2.0)	1.00
CHF, n (%)	6 (2.6)	7 (2.7)	1.00
IHD, n (%)	31 (13.5)	40 (15.7)	0.522
A-fib, n (%)	4 (1.7)	5 (2.0)	1.00
MI, n (%)	4 (1.7)	2 (0.8)	0.429
CKD, n (%)	5 (2.2)	12 (4.7)	0.146
Number of comorbidities, n (%)			0.035
0	12 (5.2)	2 (0.8)	
1	20 (8.7)	12 (4.7)	
2	50 (21.7)	61 (23.9)	
3	117 (50.9)	133 (52.2)	
4	25 (10.9)	40 (15.7)	
5	5 (2.2)	6 (2.4)	
6	1 (0.4)	1 (0.4)	
HbA1C (%, mean years ± SD)	7.7 ± 1.7	7.9 ± 1.5	0.102
T4 (pmol/L, mean years ± SD)	14.8 ± 2.4	14.9 ± 2.6	0.477
TSH (uIU/mL, mean years ± SD)	2.5 ± 1.8	2.4 ± 1.7	0.084
Serum creatinine (mcmol/L, mean years ± SD)	71.3 ± 18.9	70.4 ± 24.2	0.720
HDL (mmol/L, mean years ± SD)	1.1 ± 0.3	1.2 ± 0.3	0.293
LDL (mmol/L, mean years ± SD)	2.2 ± 0.8	2.3 ± 0.9	0.739
TG (mmol/L, mean years ± SD)	1.7 ± 0.9	1.8 ± 1.3	0.237
TC (mmol/L, mean years ± SD)	4.1 ± 0.9	4.2 ± 1.0	0.655
Bilirubin (umol/L, mean years ± SD)	7.9 ± 4.1	8.1 ± 3.6	0.918
